# Large Fragment InDels Reshape Genome Structure of Porcine Alveolar Macrophage 3D4/21 Cells

**DOI:** 10.3390/genes13091515

**Published:** 2022-08-24

**Authors:** Xiaolong Li, Xiaoqian Zhang, Yandong Luo, Ru Liu, Yan Sun, Shuhong Zhao, Mei Yu, Jianhua Cao

**Affiliations:** 1 Key Laboratory of Agricultural Animal Genetics, Breeding and Reproduction of Ministry of Education, Huazhong Agricultural University, Wuhan 430070, China; 2 College of Animal Science and Technology, Huazhong Agricultural University, Wuhan 430070, China; 3 The Cooperative Innovation Center for Sustainable Pig Production, Swine Breeding and Reproduction Innovation Platform, Huazhong Agricultural University, Wuhan 430070, China; 4 3D Genomics Research Center, Huazhong Agricultural University, Wuhan 430070, China

**Keywords:** 3D4/21 cell, structure variation, InDels, alveolar macrophages, pig

## Abstract

The porcine monomyeloid cell line, or 3D4/21 cells, is an effective tool to study the immune characteristics and virus infection mechanism of pigs. Due to the introduction of the neomycin resistance gene and the SV40 large T antigen gene, its genome has undergone essential changes, which are still unknown. Studying the variation in genome structure, especially the large fragments of insertions and deletions (InDels), is one of the proper ways to reveal these issues. In this study, an All-seq method was established by combining Mate-pair and Shotgun sequencing methods, and the detection and verification of large fragments of InDels were performed on 3D4/21 cells. The results showed that there were 844 InDels with a length of more than 1 kb, of which 12 regions were deletions of more than 100 kb in the 3D4/21 cell genome. In addition, compared with porcine primary alveolar macrophages, 82 genes including the *CD163* had lost transcription in 3D4/21 cells, and 72 genes gained transcription as well. Further referring to the Hi-C structure, it was found that the fusion of the topologically associated domains (TADs) caused by the deletion may lead to abnormal gene function. The results of this study provide a basis for elaborating the genome structure and functional variation in 3D4/21 cells, provide a method for rapid and convenient detection of large-scale InDels, and provide useful clues for the study of the porcine immune function genome and the molecular mechanism of virus infection.

## 1. Introduction

As one of the most widely farmed agricultural animals, pigs are very similar to humans compared to other non-primate mammals in anatomical structure, physiological metabolism, genetic level, and disease occurrence [1,2]. Based on these, pigs are not only suitable as a biomedical model to study human diseases [3,4,5,6], but they also have a perfect application prospect in the field of xenotransplantation [7,8,9]. As an important therapeutic target for many human diseases, macrophages are widely distributed in different tissues and organs, play a vital role in many biological processes, and show great functional diversity [10,11,12]. Compared with macrophages of other tissues, alveolar macrophages are regarded as the first important line of defense against the invasion of external microorganisms due to their unique location [13,14]. At the same time, porcine alveolar macrophages are a kind of innate immune cell in lung tissue that can secrete cellular active substances and play an important role in the immune, repair, and regeneration processes and in the maintenance of lung homeostasis [15,16,17].

Genetic variation exists widely among different species and is an important source of phenotypic diversity. Studies on genetic variation mainly focus on Single-Nucleotide Polymorphism (SNP) and structural variation (SV). Structural variation is widely distributed in the genome and is usually defined as changes in the genome sequence longer than 50 bp, including deletions, duplications, insertions, inversions, and translocations [18,19]. SV involves a longer genome sequence than SNP and therefore SV may have a greater impact on gene expression [20,21]. Studies have shown that structural variation, as an important source of driving genetic variation, directly or indirectly affects gene expression, leading to phenotypic variation and disease occurrence [22,23]. In recent years, research on the structural variation in porcine alveolar macrophages has mostly consisted of comparative analyses among breeds, but the research on the structural variation in porcine alveolar macrophages is very limited [24,25,26]. Zhou et al. found a specific SV region on the X chromosome spanning 30 Mb in Asian domestic pigs by further analysis of the newly assembled Meishan pig genome [27]. Liu et al. analyzed the structural variation in the litter sizes of Xiang pigs and identified 4637 and 4119 unique structural variations in the high-litter array and the low-litter array, respectively, and the related genes involved in these SVs are related to the reproductive traits of pigs [28].

Since the shotgun was used to sequence viral genomes in 1981 [29], whole-genome sequencing and metagenomics research have been greatly improved over the past few decades. The genomic DNA were randomly sheared and cloned into vectors, and the short-read sequencing was performed and then assembled the long fragments by the overlapping strategy, which takes advantages of proofreading for single-base and possess shortness for whole-genome assembling as well. On the contrary, the mate-pair sequencing adopts the strategy of constructing a longer fragments library and circular sequencing, which is helpful to assemble larger contigs and scaffolds but is not good at base error correction. Combining the two methods can benefit the study of genome assembly, structural variation, and diagnosis of clinical diseases and cancers [30,31].

In this study, the All-seq method combining Mate-pair and Shotgun was used to explore the structural variation in 3D4/21 cells. The reliability and accuracy of this method were verified by the library quality identification and successful verification of detected structural variation. Our research shows that there is a close relationship between the structural variation in 3D4/21 cells and its gene function, which lays a foundation for the genomic study of porcine alveolar macrophages associated with SV and provides a new basis for pig genetics and breeding.

## 2. Materials and Methods

### 2.1. All-Seq Library Preparation

An amount of 3 μg high-quality genomic DNA was set up for Tn5 tagmentation reaction followed by strand displacement (Transgen, Beijing, China). The DNA fragments were conducted to size selection by SageELF (Sage Science, Beverly, MA, USA), and the favorite sizes were collected for further use. The circularization followed end-repair, and A-tailing of linear DNA was prepared at 0.1 ng/μL in reaction buffer with 0.1 U/μL T4 DNA ligase (ABclonal, Wuhan, China) at 16 °C overnight. The circle DNA was processed with 1U/uL DNase at 37 °C for 30 min to remove linear DNA and then was sheared with sonication (Sonics, Newtown, CT, USA) to obtain 300 bp fragments. The short fragments were prepared Illumina-compatible DNA library with NEBNext Ultra II kit (NEB, Ispawich, MA, USA) according to the manufacturer’s guide. The 300~500 bp All-seq library was submitted to HiSeq3000 platform sequencing after PCR cleanup and size selection by Blue Pippin (Sage Science, Beverly, MA, USA).

### 2.2. Cell Culture and PAMs Collection

The trachea of freshly slaughtered pigs were immediately ligated, and the lungs were aseptically removed. The outer surface of the lungs was cleaned with normal saline, and 150–200 mL of PBS was injected into the lungs from the trachea. The lavage solution was recovered after 1–2 min, and the above operations were repeated until the lavage solution was clarified. The lavage solution was centrifuged at 1500 r/min for 10 min and porcine alveolar macrophages (PAM) were collected. These were washed twice and cryopreserved.

3D4/21 cells were bought from American Type Culture Collection (ATCC). 3D4/21 cells and PAMs were cultured in a complete medium composed of RPMI-1640 (Gibco, Carlsbad, CA, USA), 10% fetal bovine serum (Gibco, Carlsbad, CA, USA), 1% MEM Non-Essential Amino Acids (Gibco, Carlsbad, CA, USA), 100 U/mL penicillin, and 100 μg/mL streptomycin (Hyclone, Logan, UT, USA). Cell lines were cultured at 37 °C in a standard tissue culture incubator with 5% CO_2_.

### 2.3. DNA Extraction

Genomic DNA of 3D4/21 cells was extracted by the TIANamp Genomic DNA kit (TIANGEN, Beijing, China). Genomic DNA of Duroc, Meishan, and Yorkshire pigs were extracted from the blood sample by TIANamp Genomic DNA kit (TIANGEN, Beijing, China).

### 2.4. Identification of Large InDels

The large InDels with a length above 1 kb were identified based on mate-pair (MP) spans. According to the quartiles and interquartile range (IQR) of All-seq MP reads, the data below Q1 − 1.5 × IQR or above Q3 + 1.5 × IQR are significantly abnormal. In addition, the thresholds of large InDels were set to 4.8 kb (Q1 − 1.5 × IQR) and 7.2 kb (Q3 + 1.5 × IQR), respectively. The MP reads with a span greater than 7.2 kb were considered to be effectively large fragment deletions (red pair in browser) and vice versa for insertion (blue pair) if they only spanned less than 4.8 kb. The reads with spans between 4.8 kb to 7.2 kb were normal mate-pair reads, which implied no significant large InDels were detected in this region.

### 2.5. InDels Validation by PCR and Sanger Sequencing

The predicted SVs were verified using a pairwise PCR approach. PCR products that were successfully amplified using primers were sequenced by the Sanger technology to define the exact base sequence. A total volume of 20 μL was used for PCR and contained 1 μL of genomic DNA (1 ng/μL), 10 μL of 2× M5 HiPer plus Taq HiFi PCR Mix (with blue dye) (Mei5bio, Beijing, China), 0.5 μL of 10 μM primers, and 8 μL of ultrapure water. The PCR program was set at 95 °C for 3 min; 30 cycles of 94 °C for 25 s, 60 °C for 25 s, and 72 °C for 18 s; and a final extension of 5 min at 72 °C. The PCR products were separated on 2% agarose gels and visualized and recorded under UV light.

### 2.6. RNA-Seq

3D4/21 cells and PAMs total RNA were extracted using the RNeasy Mini kit (Qiagen, Valencia, CA, USA) following the manufacturer’s protocol and treated with DNase I (Qiagen, Valencia, CA, USA). cDNA was prepared by RNA reverse transcription using ABScript III RT Master Mix (ABclonal, Wuhan, China). The quality of RNA was detected, and RNA samples with high quality were selected (RIN ≥ 7). RNA-Seq library was prepared using a Stranded mRNA sample preparation kit (Illumina, San Diego, CA, USA) and was sequenced on the Hiseq3000 (PE150) platform.

### 2.7. Hi-C

The methods of in situ Hi-C in this study were performed as previously described in the in situ Hi-C protocol [32], with a minor modification. Briefly, one to two million cells were crosslinked with 1% formaldehyde for 10 min and quenched with 200 mM glycine for 10 min at RT. The cell lysis followed the existing protocol. The nuclear membrane was permeated by 50 μL of 0.5% SDS and quenched by 25 μL 10% TritonX-100. Chromatin followed by 100 U Mbol (NEB, Ispawich, MA, USA) digestion overnight, biotin-14-dATP (Invitrogen, Carlsbad, CA, USA) was introduced into end-blunting and in situ proximity ligation overnight using T4 DNA ligase (ABclonal, Wuhan, China). Proximal ligation DNA was obtained by de-crosslink using protein K (Invitrogen, Carlsbad, CA, USA) digestion at 55 °C overnight and Phenol: Chloroform: Isoamyl Alcohol (25:24:1) (Coolaber, Beijing, China) extraction. The biotinylated DNA was pulled down by T1 beads (Invitrogen, Carlsbad, CA, USA). In situ Hi-C library was constructed using FS DNA library prep kit (ABclonal, Wuhan, China) following the manufacturer’s instructions and was sequenced on the DNBSEQ-T7 platform.

## 3. Results

### 3.1. All-Seq Methodology

**Figure 1:**
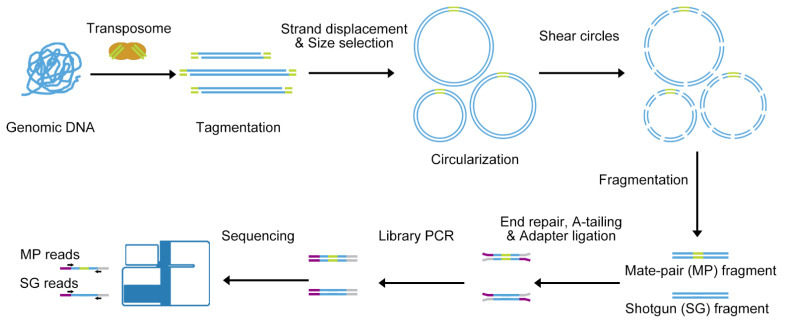
All-seq overview. Genomic DNA fragments are indicated in blue, Tn5 adapter sequence in green, P5 adapter in purple, and P7 adapter in gray. MP for Mate-pair reads and SG for Shotgun reads.

We established the All-seq method by adopting the strategy of Mate-pair and Shotgun combination (Figure 1), which can detect long fragment variations and can also detect small variations such as SNP through Shotgun. This method was used to detect structural variations in porcine alveolar macrophages (3D4/21). Firstly, genomic DNA was fragmented by Tn5 transposons and the adapter was inserted simultaneously. These fragments are then circularized, and then the resulting circles are fragmented to generate Mate-pair fragments with an adapter connection and Shotgun fragments without an adapter connection. Finally, all fragments were used for library construction and sequencing.

### 3.2. Quality Identification of All-Seq Library

**Figure 2:**
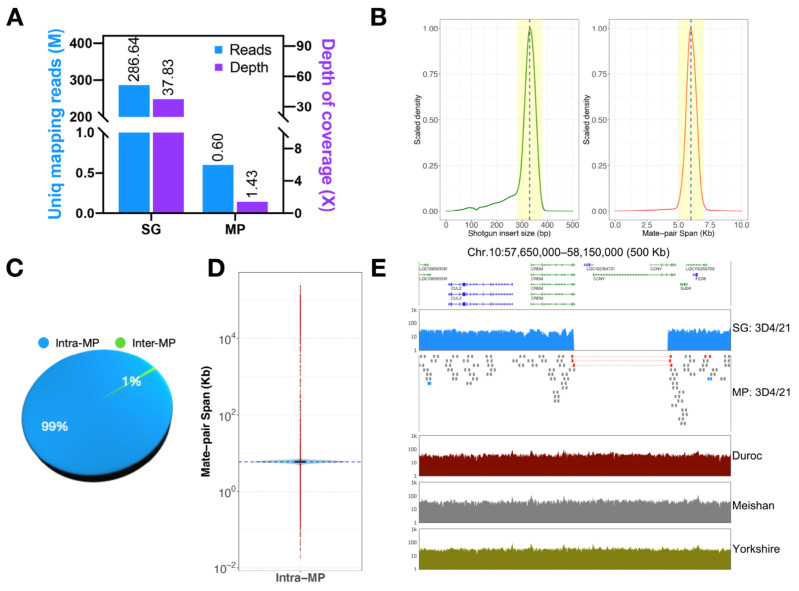
Characterization of All-Seq library of porcine macrophages (3D4/21). (**A**) The histogram shows the unique mapping reads and depth of coverage of Shotgun and Mate-pair. Blue means unique mapping reads, and purple means depth of coverage. (**B**) The scaled density plot shows the size distribution of Shotgun (left), where the dotted line represents the median 330 bp and the yellow shadow represents the median distance ±50 bp, and the span distribution of Mate-pair (right), where the dotted line represents the median 6 kb and the yellow shadow represents the median distance ±1 kb. (**C**) Pie chart showing the proportion of Intra/Inter Mate-pair. Intra-MP in blue and Inter-MP in green. (**D**) Violin plots depicting the span distribution of Intra Mate-pair. The blue highlight indicates that if the normal MP size ranges from 4.8 kb to 7.2 kb. If (Q1 − 1.5 × IQR) < 4800 bp it is defined as insertion, and if (Q3 + 1.5 × IQR) > 7200 bp it is defined as deletion. (**E**) Visual integration showing 3D4/21 cells specific structural variation on Chr.10: 57,650,000–58,150,000. Red in MP means deletion and blue means insertion. The data tracks from top to bottom show the Shotgun and Mate-pair of 3D4/21 cells, the Shotgun of Duroc, Meishan, and Yorkshire.

By analyzing the sequencing data in Table S1, it can be seen that the unique mapping reads of Shotgun are 286.64 Mb with a depth of coverage of 37.83×, while the unique mapping reads of Mate-pair are 0.6 Mb with a depth of coverage of 1.43× (Figure 2A). It indicates that our sequencing depth and data coverage are sufficient to support the detection of structural variation. We also observed that most (85%) Shotgun sizes are around 330 bp, which is effective for detecting small variation. However, the span of the Mate-pair (49%) is concentrated at about 6 kb (Figure 2B), indicating that it plays a major role in inferring the information of structural variation in the whole region between the two paired ends. A total of 586,071 MPs were identified, including 581,598 Intra-MP and 4473 Inter-MP (Figure 2C), indicating that the vast majority (99%) of MPs were only involved in insertions, deletions, repetitions, and inversions within the same chromosome and very few (1%) were related to translocations between different chromosomes. Further analysis of the span of Intra-MP found that 580,754 MP sizes ranged from 4.8 to 7.2 kb, 418 were smaller than 4.8 kb, and 426 were larger than 7.2 kb (Figure 2D). We also list the top 10 lengths for deletions and insertions in Table S2. By mapping Mate-pairs and Shotguns together to the pig reference genome (Sscrofa11.1), we were able to identify the specific structural variation in 3D4/21 cells well, including deletions and insertions (Figure 2E). The results showed that this method was effective for detecting structural variation.

### 3.3. Detection of Structural Variation in 3D4/21 Cells

**Figure 3:**
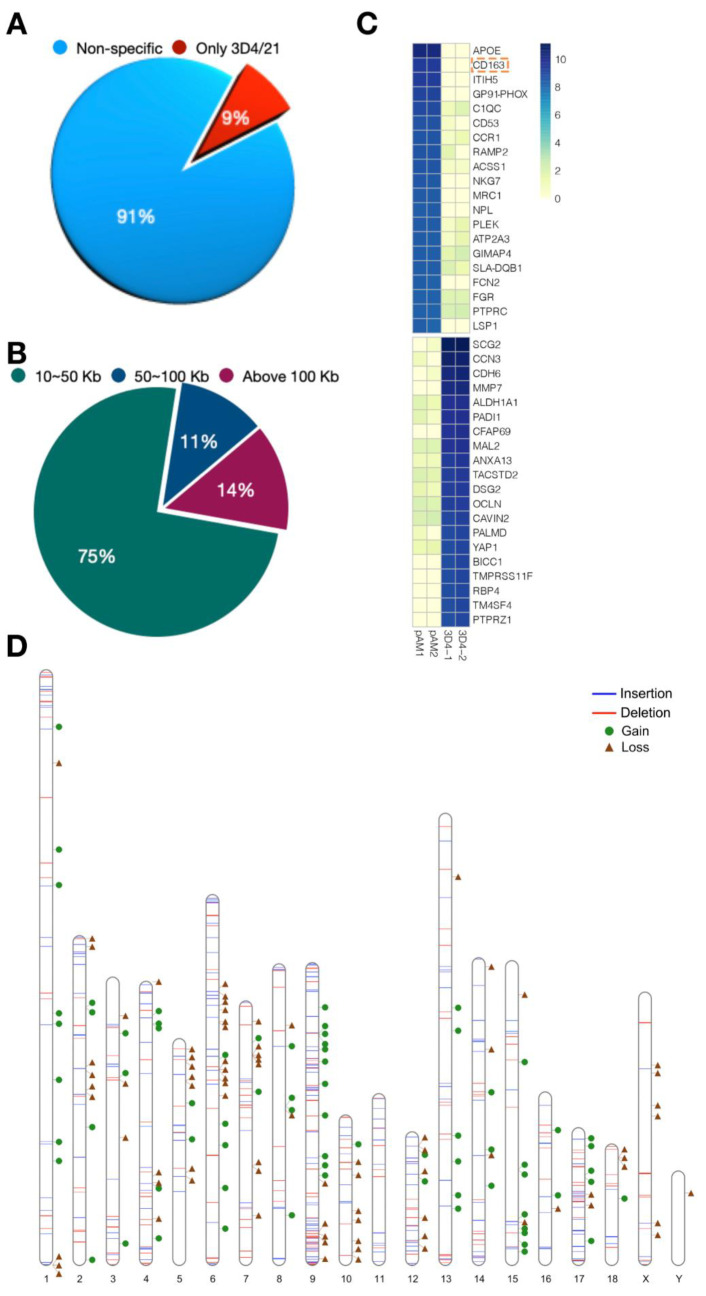
The size and distribution of structural variation in 3D4/21 cells. (**A**) The pie chart shows the relative proportion of 3D4/21 cells specific deletions over 10 kb. (**B**) Pie chart showing the subdivision of the specific deletions of 3D4/21 cells over 10 kb. (**C**) The volcano diagram shows the top 20 gene loss and gain of 3D4/21 cells relative to PAMs. (**D**) SV distribution on different chromosomes. White bars represent all chromosomes. Blue lines represent insertion, red lines represent deletion, green circles represent gene gain, and brown triangles represent gene loss.

Deletion is one of the most studied types of structural variation. A total of 19,585 deletions larger than 1 kb were identified in 3D4/21 cells by the All-seq strategy, and 971 were larger than 10 kb. Since the structural variation of large fragments may have a greater impact on gene expression, we selected deletions larger than 10 kb for in-depth study and found that only 87 (9%) of them were unique to 3D4/21 cells (Figure 3A). Furthermore, we divided the deletions larger than 10 kb that were unique to 3D4/21 cells. It can be seen that 65 (75%) ranged between 10 kb and 50 kb, 10 (11%) ranged between 50 kb and 100 kb, and 12 (14%) were larger than 100 kb (Figure 3B), and these unique deletions were speculated to be related to their gene functions. By analyzing transcriptomic data of PAMs and 3D4/21 cells to explore gene expression, we selected the top 20 genes with the largest difference between PAMs and 3D4/21 cells by the RKPM data of RNA-seq and showed them in the heat map (Figure 3C). Interestingly, there are many immune-related genes in the deletion and acquisition of 3D4/21 cells, such as *CD163*, one of the cellular receptors for porcine reproductive and respiratory syndrome virus (PRRSV), which is also involved in immune signaling. Next, we analyzed the distribution of SV on chromosomes and combined them with the positions of loss and gain genes. It could be seen that structural variation was closely related to gene function (Figure 3D).

### 3.4. Validation of Structural Variation in 3D4/21 Cells

**Figure 4:**
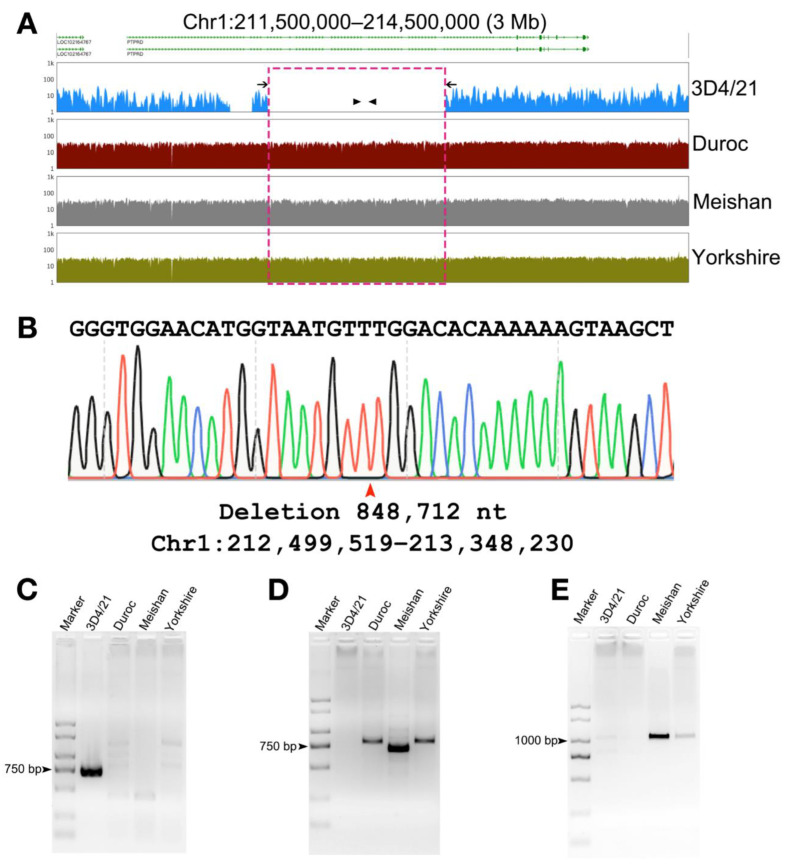
Schematic diagram of structural variation in 3D4/21 cells. (**A**) Visual integration for Chr1: 211,500,000–214,500,000 in 3D4/21 cells, showing examples of deletions. The dashed box highlighted indicates the deletion location. (**B**) Base sequence analysis showed the exact deletion sites (red arrow). Length: 848,712 nt, Region: Chr1: 212,499,519–213,348,230. (**C**) PCR amplification of 3D4/21 cells’ specific deletions, with primer (F1/R1) locations corresponding to black arrow in (**A**). Marker, DL2000. Lanes 2–5 correspond to 3D4/21 cells gDNA, Duroc gDNA, Meishan gDNA, and Yorkshire gDNA, respectively. (**D**) PCR amplification of 3D4/21 cells’ specific deletions, with primer (F2/R2) locations corresponding to the black triangle in (**A**). Lanes 1–5 are the same as above. (**E**) PCR amplification of 3D4/21 cells insertion sites. Lanes 1–5 are the same as above.

To verify the identified SV, we extracted genomic DNA from 3D4/21 cells and different pig breeds. We then selected Chr1:211,500,000–214,500,000 deletion sites specific to 3D4/21 cells for PCR amplification (Figure 4A), and detailed primer sequences (F1/R1 and F2/R2) are listed in Table S3. When amplifying from the external ends of the deletion site, agarose gel electrophoresis results showed that there was a band in 3D4/21 cells, while there were no bands in Duroc, Meishan, and Yorkshire because the amplified fragment was too large (Figure 4C). There was no band in 3D4/21 cells during the amplification inside the deletion site, and there were bands in Duroc, Meishan, and Yorkshire because there was no primer binding site in 3D4/21 cells (Figure 4D). We then performed Sanger sequencing analysis on the amplified PCR products and obtained the specific deletion size and site (Figure 4B). PCR validation of the 3D4/21 cells insertion site was also shown (Figure 4E), and detailed primer sequences (F3/R3) are shown in Table S3.

### 3.5. Structural Variation in the Genome Can Cause Changes in the Three-Dimensional Structure of Chromatin and Thus Affect Gene Transcription

**Figure 5:**
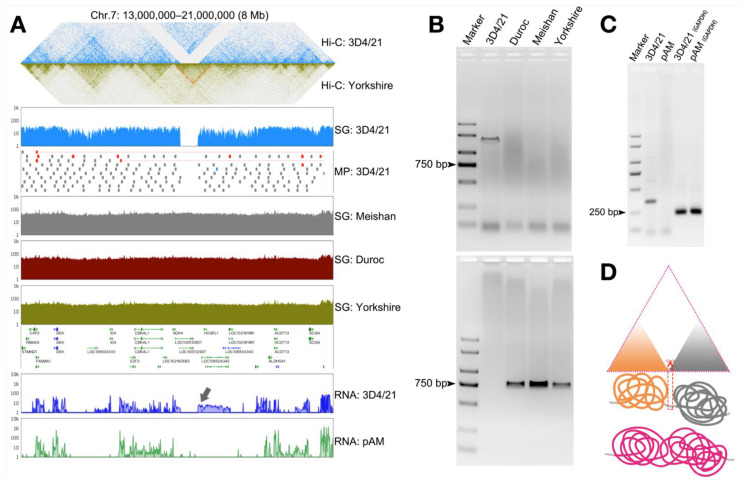
The effects of structural variation in 3D4/21 cells. (**A**) Visual integration for Chr7: 13,000,000–21,000,000 in 3D4/21 cells, showing the example of deletion leading to changes in the three-dimensional structure of chromatin and thus affecting gene transcription. Orange highlights changes in the 3D chromatin structure of 3D4/21 cells relative to Yorkshire pigs. The black arrow points to changes in 3D4/21 cells relative to the PAMs transcript. (**B**) 3D4/21 cells’ specific deletion in (**A**) was verified by PCR amplification, with primer locations corresponding to external ends of the deletion site (top) and inside of the deletion site (bottom). Lanes 1–5 are the same as above. (**C**) PCR amplification was used on cDNA to verify the difference between the 3D4/21 cells and PAMs transcripts, with primer (F6/R6) locations corresponding to the gray arrow in (**A**). Marker, DL2000. Lanes 2–3 correspond to transcription amplification of selected locations of 3D4/21 cells cDNA and PAMs cDNA. Lanes 4–5 correspond to *GAPDH*. The detailed primer sequences are shown in Table S3. (**D**) The schematic of deletion reshapes the genome structure of 3D4/21 cells. Red scissors correspond to the deletion position, orange and gray mean different TAD, and purple means fused TAD.

To further explore the effects of SV in 3D4/21 cells, we combined Hi-C and RNA-seq data and found that the specific deletion in 3D4/21 cells in Chr7: 13,000,000–21,000,000 resulted in changes in the three-dimensional structure (TAD) of chromatin and affected gene transcriptional expression at this site (Figure 5A). The PCR amplification was also used to verify the deletion sites in 3D4/21 cells (Figure 5B), and detailed primer sequences (F4/R4 and F5/R5) are shown in Table S3. At the same time, total RNA of 3D4/21 cells and PAMs were extracted and reverse transcribed, and the changes in the transcript were verified by cDNA obtained by PCR amplification (Figure 5C). It was found that the specific deletions in 3D4/21 cells led to increased transcription of downstream genes, and we proposed a mechanism hypothesis that the deletion may reshape the genomic structure of 3D4/21 cells, resulting in TAD fusion on both sides of the deletion site (Figure 5D). This may lead to abnormal transcription initiation, resulting in pseudogene transcription.

## 4. Discussion

Structural variation can affect the expression of related genes to a certain extent. It has been reported that SV can affect the molecular mechanism of gene regulation by disrupting the three-dimensional structure of chromatin and further causing diseases [33]. At the same time, a large number of studies have shown that the structural variation in the genome is associated with a variety of diseases, including autism, schizophrenia, and intellectual disabilities [34,35]. Notably, mutations in the structure of the genome may increase the body’s susceptibility to disease, resulting in large-scale transcriptome sequence changes commonly associated with cancer [36,37,38]. Therefore, the study of structural variation is of great significance for clinical treatment. The All-seq method provided in this study can quickly and conveniently detect the InDels of large fragments, laying a foundation for the in-depth study of structural variation.

Insertions and deletions that focus on characterization in our study are usually associated with gene function. We found that 3D4/21 cells lost transcription of 82 genes, including *CD163*, and gained 72 genes compared to PAMs. When we combined the analysis of SV distribution and the location of gene gain and loss on the 3D4/21 cell chromosome, it was found that there was a close relationship between structural variation and gene function, which was consistent with previous reports. It has been found that the deletion of 281 bp in the first intron of *MYL4* in Ningxiang pigs promoted the formation of subcutaneous fat [39], and the deletion of the intron of *IGF2R* in Tibetan pigs inhibited its growth rate [27]. By further combining Hi-C data, we found that the deletion of large fragments resulted in the change in 3D genomic domains in 3D4/21 cells, leading to gene dysfunction. We inferred that the reason might be TAD fusion caused by deletions, which led to enhancer adoption. The specific mechanism needed to be further explored.

With the continuous development and progress of sequencing technology, the characterization of pig structural variation is gradually improved, and more and more discoveries show that structural variation is associated with pig disease resistance mechanisms and genetic breeding. It has been reported that the high fertility of the Meishan pig may be related to its unique SV in the comparison between Meishan and Duroc pigs in large-scale population resequencing [40]. The *ZC3H12B* gene annotated in the SV hotspot region found on the X chromosome of Guizhou indigenous pigs may be associated with host immunity and inflammatory diseases [25]. In addition, Yang et al. revealed the genetic differences of Chinese pigs through a comprehensive analysis of the SV in Chinese pig breeds [24]. Therefore, the structural variation in different breeds of pigs may be the key factor affecting the reproduction, metabolism, immunity, and growth and development of pigs, and the mechanism of action still needs to be further explored. In this study, InDels in 3D4/21 cells were detected, which not only explained the structural and functional variation in the genome but also provided useful clues for the study of pig immune function genome and molecular mechanisms of virus infection.

## Data Availability

The sequencing data in this article can be available upon request for research purposes. This paper also analyses existing, publicly available data. The source for these datasets is listed in Table S1.

## Supplementary Materials

The following supporting information can be downloaded at: https://www.mdpi.com/article/10.3390/genes13091515/s1, Table S1: The data source in this study. Table S2. Deletion and insertion of the top 10 lengths. Table S3: All primers used in this study.
