# Supplementary material for: Large Fragment InDels Reshape Genome Structure of Porcine Alveolar Macrophage 3D4/21 Cells
Source: Genes (Basel). 2022 Aug 24;13(9):1515. doi: 10.3390/genes13091515 (PMC9498719; doi:10.3390/genes13091515)
Supplement: Supplementary file 1 [file genes-13-01515-s001.zip › genes-1843157-supplementary Tables S1¿CS3.pdf]

Table S1. The data source in this study.

DATA	SOURCE	IDENTIFIER
MP: 3D4/21	3D4/21 Mate-pair sequencing data	This study
SG: 3D4/21	3D4/21 Shotgun sequencing data	This study
SG: Meishan	Meishan Shotgun sequencing data	This study
SG: Yorkshire	Yorkshire Shotgun sequencing data	This study
SG: Duroc	Duroc Shotgun sequencing data	Reference (BioProject: PRJNA597497)
RNA-seq: 3D4/21	3D4/21 RNA-seq sequencing data	This study
RNA-seq: PAM	PAM RNA-seq sequencing data	This study
Hi-C: 3D4/21	3D4/21 Hi-C sequencing data	This study
Hi-C: Yorkshire	Yorkshire Hi-C sequencing data	Reference (BioProject: PRJNA597497)

Table S2. Deletion and insertion the top 10 length.

Chr	Start	End	Length (nt)	Type
1	212,715,841	213,196,653	480,812	Deletion
7	17,073,787	17,329,408	255,621	Deletion
7	17,329,694	17,529,750	200,056	Deletion
10	57,897,808	58,049,063	151,255	Deletion
1	5,841,223	5,987,524	146,301	Deletion
5	104,374,167	104,507,956	133,789	Deletion
1	200,744,065	200,863,288	119,223	Deletion
8	126,425,446	126,532,507	107,061	Deletion
5	97,725,694	97,822,976	97,282	Deletion
17	23,584,152	23,642,742	58,590	Deletion
2	45,606,489	45,607,345	4,100	Insertion
2	102,080,108	102,081,987	3,119	Insertion
5	39,798,025	39,799,042	3,911	Insertion
6	170,070,731	170,071,726	3,959	Insertion
9	44,212,249	44,214,070	3,124	Insertion
9	63,377,248	63,377,737	4,458	Insertion
10	39,875,500	39,875,792	4,615	Insertion
11	72,861,079	72,862,086	3,971	Insertion

12	19,549,935	19,551,398	3,486	Insertion
13	180,943,835	180,944,946	3,820	Insertion

Table S3. All primers used in this study.

Primer	Sequence(5'-3')
F1	GGTTGGGAGAAAGCCTATTTCA
R1	AGTACAGAGGTGTGCCTTTAC
F2	TCTGATTCAGGCACAAGCCCT
R2	CTGCACTGTTGTTCAAAGGCA
F3	CGACTTTGCTGATGAATTGTTCCA
R3	GGGTTGCTTTTATTTCTGGGGT
F4	GGGTTGCTTTTATTTCTGGGGT
R4	ACCTTGTAAGTAAGTGGCACCT
F5	ATAGCAATCAAGCCACCCTT
R5	GCAGCTGTTCATTTCGAGCT
F6	ATGCTCTTGCATCAGTTGGGA
R6	ATCTAGTCACTGATGGCACTCT
F-GAPDH	GGCGTGAACCATGAGAAGTATG
R-GAPDH	GGTAGAAGCAGGGATGATGTTC
